# Supplementary material for: Modeling Multivariate Distributions of Lipid Panel Biomarkers for Reference Interval Estimation and Comorbidity Analysis
Source: Healthcare (Basel). 2025 Oct 1;13(19):2499. doi: 10.3390/healthcare13192499 (PMC12523935; doi:10.3390/healthcare13192499)
Supplement: Supplementary file 1 [file healthcare-13-02499-s001.zip › healthcare-3868260-supplementary.pdf]

Supplementary Information

Supplementary Information to Modeling Multivariate Distributions of Lipid Panel Biomarkers for Reference Interval Estimation and Comorbidity Analysis

Julian Velev [1,2,*], Luis Velázquez-Sosa [3], Jack Lebien [2], Heeralal Janwa [4] and Abiel Roche-Lima [3]

[1] Department of Physics, University of Puerto Rico, Puerto Rico, PR 00925-2537, USA
[2] Abartys Health, San Juan, PR 00907-3913, USA; jlebien@abartyshealth.com
[3] Center for Collaborative Research in Health Disparities, RCMI Program, Medical Science Campus, University of Puerto Rico, San Juan, PR 00936-5067, USA; luisfernandojavier.velazquez@upr.edu (L.V.-S.); abiel.roche@upr.edu (A.R.-L.)
[4] Department of Mathematics, University of Puerto Rico, Puerto Rico, PR 00925-2537, USA; heeralal.janwa@upr.edu
* Correspondence: julian.velev@upr.edu

1. Methods

1.1. Data processing

All available laboratory results for the analytes of interest (lipid panel and comorbidity markers) were extracted by LOINC code. Each record contained a non-PII person identifier, sex, age at the time of testing, and the test date. After loading the raw data, we removed physiologically impossible values (i.e., values incompatible with life), which are typically attributable to coding or unit errors. To reduce repeated-measure noise and account for clustered visits, we coarse-grained the data by calendar month: for each individual and LOINC, all results within a given month were averaged to yield a single representative value. The resulting tables were then joined across LOINCs using the combination of person ID, year, and month as keys, producing aligned multivariate records for each person–month. This workflow is summarized in Figure S1a.

1.2. Model fitting

Population segmentation was performed by iterating over demographic groups defined by sex and age. For each segment, we subset the joined dataset to obtain the corresponding multivariate sample. Within each subset, variable-appropriate transformations (e.g., log for positively skewed analytes) were applied, followed by multivariate BGMM fitting to estimate component weights, means, and covariances. The resulting per-segment mixture components were then assembled into a population-level biomarker distribution file, ordered by component weight, providing the basis for age- and sex-specific RIs and percentile-based interpretation of individual results. This process is illustrated in Figure S1b.

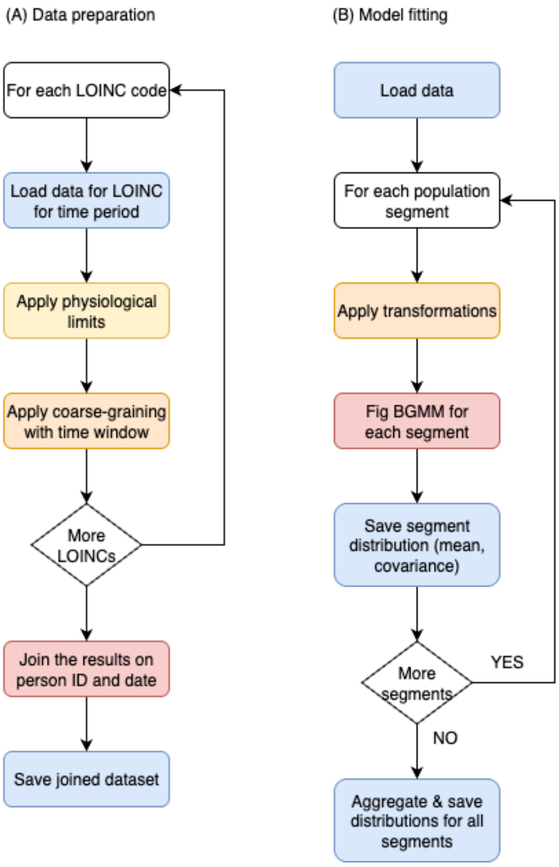

Figure S1 Workflow overview: (a) data preprocessing from raw laboratory results to aligned person–month records, and (b) Bayesian Gaussian mixture model fitting across demographic segments.

1.3. Inference

From the fitted population distributions, the Gaussian component corresponding to the healthy population can be used to construct reference surfaces (RS) across multiple biomarkers or reference intervals (RI) in one dimension. For a given central percentile (typically the 95% interval), the RS/RI defines the expected range of values for a healthy population, stratified by age and sex as illustrated in Figure S2a. This provides a standardized benchmark for assessing whether an individual result falls within normal limits.

Alternatively, given a set of biomarker results, one can compute the corresponding percentile within the population distribution. This yields a continuous measure of abnormality or risk, rather than a binary "within/outside RI" flag as shown in Figure S2b. Percentile-based interpretation allows more nuanced assessment, highlighting degrees of deviation from the expected distribution that may be clinically relevant even if results remain technically within the conventional RI.

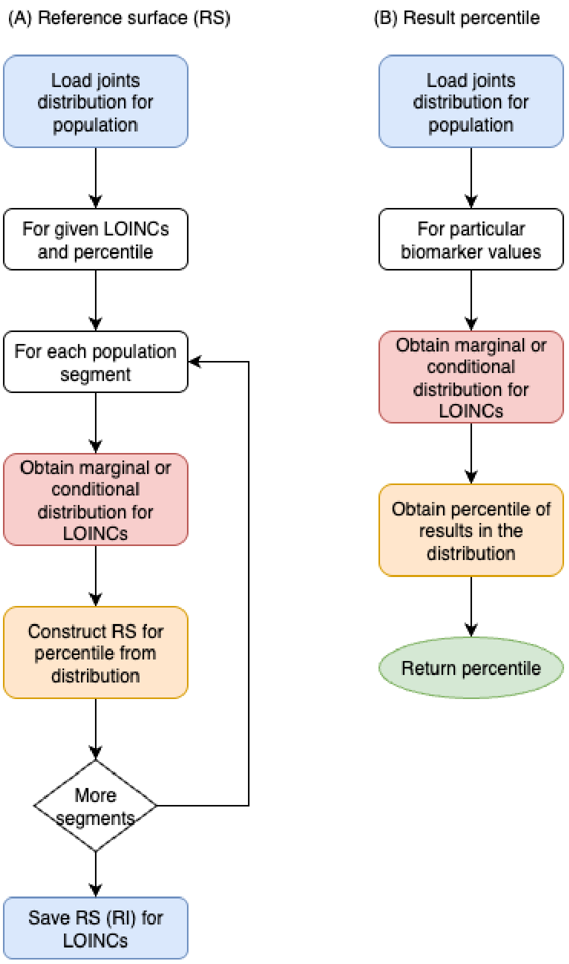

Figure S2 Inference workflows: (A) construction of reference surfaces (RS) or one-dimensional reference intervals (RI) from the healthy population component, and (B) computation of percentiles for individual biomarker results within the population distribution.

2. Results

2.1. Implication network

To better understand how comorbidities influence lipid panel biomarkers, we constructed a condition implication network. First, diagnostic criteria were defined for each condition of interest (Table 2) based on established clinical thresholds. These rules were applied to the joined biomarker dataset (linked by patient ID and date), yielding a binary diagnosis matrix where each row corresponds to a patient–month and each column to a condition.

From this binary matrix, we computed pairwise conditional probabilities of the form P(B | A), representing the probability of condition B being present given condition A. These values form the implication matrix, which is generally asymmetric (P(B | A) $\neq$ P(A | B)), allowing identification of directional relationships. Strong implications (high P(B | A)) were interpreted as potential comorbidity pathways or progression patterns. The resulting implication matrix was visualized both as a heatmap (Figure S3) and as a directed network, where edges represent the strength and direction of implication. This framework highlights not only co-occurrence but also asymmetry in disease associations, providing insight into precursor–complication relationships among cardiometabolic conditions.

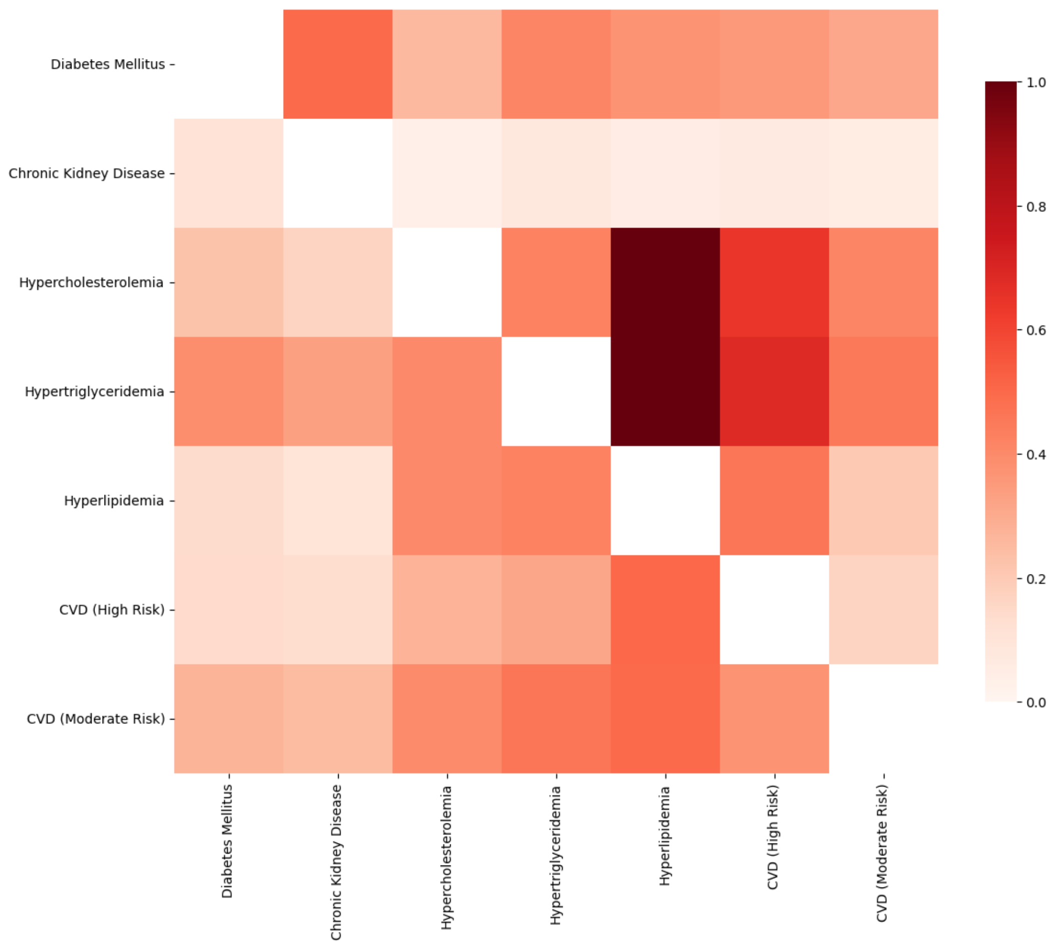

Figure S3 Heatmap of the comorbidity implication matrix, showing conditional probabilities P(B|A) for all pairs of conditions. Warmer colors indicate stronger directional associations, highlighting asymmetries in comorbidity relationships.
